# Supplementary material for: Acquired syphilis in older people in Brazil from 2010–2020
Source: PLoS One. 2024 Sep 6;19(9):e0296481. doi: 10.1371/journal.pone.0296481 (PMC11379233; doi:10.1371/journal.pone.0296481)
Supplement: S1 File — (PDF) [file pone.0296481.s001.pdf]

República Federativa do Brasil
Ministério da Saúde

SINAN
SISTEMA DE INFORMAÇÃO DE AGRAVOS DE NOTIFICAÇÃO
FICHA DE INVESTIGAÇÃO **SÍFILIS ADQUIRIDA**

Nº

CASO SUSPEITO DE SÍFILIS ADQUIRIDA: indivíduo com evidência clínica de sífilis e/ou sorologia não treponêmica reagente.
CASO CONFIRMADO DE SÍFILIS ADQUIRIDA: indivíduo com sorologia treponêmica reagente.

Dados Gerais

1 Tipo de Notificação — 2 - Individual

2 Agravo/doença — **SÍFILIS ADQUIRIDA** — Código (CID10) A53.9

3 Data da Notificação

4 UF

5 Município de Notificação — Código (IBGE)

6 Unidade de Saúde (ou outra fonte notificadora) — Código

7 Data do Diagnóstico

Notificação Individual

8 Nome do Paciente

9 Data de Nascimento

10 (ou) Idade — 1 - Hora 2 - Dia 3 - Mês 4 - Ano

11 Sexo — M - Masculino F - Feminino I - Ignorado

12 Gestante — 6 — 6 - Não se aplica

13 Raça/Cor — 1-Branca 2-Preta 3-Amarela 4-Parda 5-Indígena 9- Ignorado

14 Escolaridade
0-Analfabeto 1-1ª a 4ª série incompleta do EF (antigo primário ou 1º grau) 2-4ª série completa do EF (antigo primário ou 1º grau)
3-5ª à 8ª série incompleta do EF (antigo ginásio ou 1º grau) 4-Ensino fundamental completo (antigo ginásio ou 1º grau) 5-Ensino médio incompleto (antigo colegial ou 2º grau)
6-Ensino médio completo (antigo colegial ou 2º grau) 7-Educação superior incompleta 8-Educação superior completa 9-Ignorado 10- Não se aplica

15 Número do Cartão SUS

16 Nome da mãe

Dados de Residência

17 UF

18 Município de Residência — Código (IBGE)

19 Distrito

20 Bairro

21 Logradouro (rua, avenida,...) — Código

22 Número

23 Complemento (apto., casa, ...)

24 Geo campo 1

25 Geo campo 2

26 Ponto de Referência

27 CEP -

28 (DDD) Telefone

29 Zona — 1 - Urbana 2 - Rural 3 - Periurbana 9 - Ignorado

30 País (se residente fora do Brasil)

Dados Complementares do Caso

Dados clínicos e epidemiológicos

31 Ocupação

32 Antecedente de sífilis — 1 - Sim 2 - Não 9 - Ignorado

33 Se sim, o tratamento foi realizado? — 1 - Sim 2 - Não 9 - Ignorado

34 Comportamento Sexual
1 - Relações sexuais com homens 2 - Relações sexuais com mulheres
3 - Relações sexuais com homens e mulheres 9 - Ignorado

Dados clínicos e laboratoriais

Resultado dos Exames

35 Teste não treponêmico — 1-Reagente 2-Não Reagente 3-Não Realizado 9-Ignorado

36 Título — 1:

37 Data

38 Teste treponêmico — 1-Reagente 2-Não reagente 3-Não realizado 9-Ignorado

39 Classificação Clínica — 1 - Primária 2 - Secundária 3 - Terciária 4 - Latente 9 - Ignorado

Tratamento

40 Esquema de tratamento realizado
1 - Penicilina G benzantina 2.400.000 UI
2 - Penicilina G benzantina 4.800.000 UI
3 - Penicilina G benzantina 7.200.000 UI
4 - Outro esquema
5 - Não realizado
9 - Ignorado

41 Data do início do tratamento

Conclusão

42 Classificação Final do caso — 1 - Confirmado 2 - Descartado ______________

Observações adicionais

Investigador	Município/Unidade de Saúde		Cód. da Unid. de Saúde
	Nome	Função	Assinatura

OBSERVAÇÕES:
Considera-se a data de notificação como sendo data de preenchimento da ficha de notificação e a data de diagnóstico como sendo a data da coleta de material para exame laboratorial ou da evidencia clínica.

I**NSTRUÇÕES PARA O PREENCHIMENTO:** Nenhum campo deverá ficar em branco.

31 - Informar a ocupação do indivíduo no momento do diagnóstico. Refere-se à atividade exercida pelo paciente no setor formal, informal ou autônomo ou sua última atividade exercida quando paciente for desempregado. O ramo de atividade econômica do paciente refere-se às atividades econômicas desenvolvidas nos processos de produção do setor primário (agricultura e extrativismo); secundário (indústria) ou terciário (serviços e comércio).

32 - Informar se o paciente no passado já teve sífilis. O relato do paciente será considerado.

33 - Caso tenha antecedente, informar se o tratamento foi realizado.

34 - Informar o comportamento sexual.

35 - Teste de sorologia não treponêmica indicada para triagem (VDRL - Veneral Diseases Research Laboratory ou RPR - Rapid Plasma Reagin)

36 - Informar a titulação do teste VDRL ou RPR.

37 - Informar a data da coleta do teste

38 - FTA-Abs (Fluorescent Treponemal Antibody-absorption), MHA-Tp (Microhemaglutination Treponema pallidum Assay), TPHA (Treponema pallidum Hemaglutination Assay), ELISA (Enzyme-Linked Immunosorbent Assay), testes rápidos para diagnóstico de sífilis (testes imunocromatográficos).

39 - Registrar a classificação clínica para sífilis:
1 - sífilis primária - cancro duro;
2 - sífilis secundária – lesões cutâneo-mucosas (roséolas, sifílides papulosas, condiloma plano, alopécia);
3 - sífilis terciária – lesões cutâneo-mucosas (tubérculos ou gomas); alterações neurológicas (tabes dorsalis, demência); alterações cardiovasculares (aortite sifilítica, aneurisma aórtico); alterações articulares (artropatia de Charcot);
4 - sífilis latente - fase assintomática o diagnóstico apenas é obtido por meio de reações sorológicas.

40 - Esquema de tratamento:
· Sífilis primária: penicilina benzatina 2,4 milhões UI, IM, em dose única (1,2 milhão U.I. em cada glúteo).
· Sífilis secundária e latente recente: penicilina benzatina 2,4 milhões UI, IM, repetida após 1 semana. Dose total de 4,8 milhões U.I.
· Sífilis tardia (latente e terciária): penicilina benzatina 2,4 milhões UI, IM, semanal, por 3 semanas. Dose total de 7,2 milhões U.I.

41 - Informar a data do início do tratamento.

42 - Informe a classificação final do caso. Considera-se caso **confirmado** o indivíduo com **sorologia treponêmica reagente**. Somente considera-se descartado com sorologia treponêmica não reagente

Para fins de vigilância no nível local atentar para:

1. Evidências de outras DST;

2. Abordagem das parcerias, visando à quebra da cadeia de transmissão, considerando abordagem consentida
