# Supplementary material for: Acquired syphilis in older people in Brazil from 2010–2020
Source: PLoS One. 2024 Sep 6;19(9):e0296481. doi: 10.1371/journal.pone.0296481 (PMC11379233; doi:10.1371/journal.pone.0296481)
Supplement: Attachment — Submitted filename: Response to reviewers.pdf [file pone.0296481.s002.pdf]

Dear Editors and Reviewers,

I greet you, thank you for your contributions, and present the responses to your requests for better adequacy of the manuscript.

To the Editors

In the Introduction section, when asked to present an overview and clear view of the epidemiology of syphilis among older people in Brazil, the 6th paragraph was restructured to meet the request to provide a clearer picture of the epidemiology of syphilis among older adults in Brazil. In addition, we updated the epidemiological data, showing the increased detection rate of acquired syphilis in individuals aged 50 years and over (lines 88-96).

Given the need to emphasize the importance of the study, a paragraph (lines 112-118) was included to demonstrate that the study's relevance lies in verifying whether the increase in syphilis also occurred among elderly individuals. The elderly population is usually forgotten when sexually transmitted infections are investigated. It is not even thought of evaluating these individuals through laboratory tests. There is structural ageism, making these people's sexuality invisible. That way, the research will be essential to print the picture of the infection in Brazil in elderly individuals. This will help to combat the spread of the disease and help outline perspectives for coping with this group.

Although the Acquired Syphilis Notification/Investigation form (used to record the identified cases) presents the question regarding the clinical classification (primary, secondary, tertiary, latent) and the performance of a non-treponemal test (1-reagent, 2-non-reactive, 3-not performed, 9-ignored), the relevant responses) are not published. Thus, the database does not provide information regarding clinical symptoms or laboratory results of serial serologies.

Thus, it was decided to replace the terms “incident” and “prevalent” with “detection rate” since the limitations proposed by what is informed in the database should be considered, whose information was constructed from what was available in the database Acquired Syphilis Notification/Investigation Form.

For better organization, the methodological section was subdivided. In addition, the number of individuals involved in the study was added as available in the database.

As suggested by reviewers, the discussion and conclusion sections have been rewritten.

The data that supported the construction of the results were available in a PDF file as supporting information.

We redid the writing review in the English language.

To Reviewer 1

1. Expanding access to the rapid syphilis test in campaigns and the routine of basic health units increased the detection rate of acquired syphilis and, consequently, the effective notification of cases of the disease (lines 319-321). Compulsory notification, as of 2010, demanded greater responsibility on the part of health units regarding the registration of syphilis cases, consequently generating an initial impact on the increase in the disease detection rate (lines 289-291). As it defines itself, the SINAN Reports program generates indicators with the SINAN system as a data source. Among the indicators, there is regularity in the supply of SINAN and those important for the management and innovation of the Unified Health System. The reports are generated from DBF bases of the NET or ONLINE versions of the system (lines 305-309). It was observed in the study that the increase in testing coverage for syphilis and the reorganization of health units did not stabilize the ascending curve of the disease (lines 321-322). In addition, in ten years of operation, it is indisputable to say that there was not enough time to guarantee compliance with the system's functioning, nor to believe that the increments of the disease are still related to the novelty of using the system.

2. Although the Notification/Investigation of Acquired Syphilis form presents a question regarding the clinical classification (primary, secondary, tertiary, latent) and another one related to the performance of a non-treponemal test (1-reactive, 2-non-reactive, 3-not performed, 9-ignored), this information is not included in the system and/or publicized. Thus, the database does not provide information

regarding clinical symptoms or laboratory results of serial serologies (lines 301-304). Therefore, it is impossible to determine the stage of the disease in which the patient is and inform conditions regarding the presence or absence of symptoms.

About the questioning of the motivation for the increase in cases of notification of acquired syphilis in the elderly, we present answers in some lines (98-103) of the Introduction and Discussion (319-324; 377-378; 380 – 381; 391-397; 403-406): the following are mentioned as the main determinants for the spread of syphilis: poor access to information; lower health coverage; increased rapid test availability; incomplete investigation or underreporting; late diagnosis; access limitations or discontinuation of treatment and age-related discrimination.

3. As already mentioned, in the National System of Compulsory Notification of cases of syphilis, clinical or laboratory data are not recorded, which prevents identifying the stage of the disease in which the patient is and monitoring the clinical evolution of the same.

The SINAN source includes only case identification data: date of diagnosis, date of notification, sex, date of birth, race, and education, and it is not possible to carry out a check on compliance with the case definition. In the notification of acquired syphilis, it would be important to discriminate the phases of the disease: primary, secondary, latent, and late, through the description of the individual's symptomatology; and present the laboratory results of serial serologies to support the precise definition of incident and prevalent cases, for studies like this one. Due to this fragility, it was decided to replace the terms “incident” and “prevalent” with detection rate when considering the limitations proposed by what is reported in the database itself.

To Reviewer 2

1. The 6th and 7th paragraphs of the introduction present information that better describe the epidemiology of syphilis in Brazil and contextualize the increase in cases of the disease in the country (lines 88-104). In addition, paragraph 9 describes the importance of the research for the literature and public health about developing strategies for coping with the disease, including older people in the potential risk group (lines 112-118).
2. All discussion has been reviewed and readjusted. Some paragraphs have been added, and others removed. We present the results found during the research and organize the section. The paragraphs that referred to the same approach were unified. Other studies were added to improve the writing and ease of the text. Unnecessary phrases have been duly removed.
3. The conclusion was rewritten to answer the proposed objectives for the execution of the study in a more precise and succinct way.

   The term "observational time series study" on page 2, line 35, was replaced by "cross-sectional time series study."

   The STROBE guideline was used to help fulfill the requirements related to the type of study carried out (sent as a supplementary file).
