# Supplementary material for: Acquired syphilis in older people in Brazil from 2010–2020
Source: PLoS One. 2024 Sep 6;19(9):e0296481. doi: 10.1371/journal.pone.0296481 (PMC11379233; doi:10.1371/journal.pone.0296481)
Supplement: Attachment — Submitted filename: Response to Reviewers.docx [file pone.0296481.s003.docx]

Dear Editors,

Below are the responses to all the considerations made by the reviewers. We would also like to thank the suggestions for improving the article. Anything that needed to be added or modified in the text is highlighted in the revised manuscript.

**Answer to Reviewer #1**

**1.**  **Discussion of low level of education as related to syphilis (357). It is not clearly that low education is causally related to syphilis or even truly associated. In order to study an association, one would have to have a comparison group and show that people with syphilis have lower educational achievement than population norms for this age in Brazil. In addition, the discussion about education has a somewhat pejorative tone "lower education level is associated with lower health concerns...."**

As suggested by the reviewer, we adjusted the text in lines 357 - 362.

**2. The conclusion (432) notes black race as associated with syphilis whereas the data (191) demonstrated slightly higher diagnoses in whites (though strikingly similar rates in white and black populations.**

Modified text for better understanding in line 436.

**3. Proof carefully for pejorative language (334) such as "men tend to be negligent with their health."**

Text rewritten to avoid misinterpretation, in lines 336 – 337.

**4.** **Finally, when discussing deaths due to syphilis, be certain that syphilis was the cause of death (as opposed to dying with untreated syphilis where syphilis was not the cause of death.**

Based on what is defined by the syphilis notification form, we readjusted the number of deaths caused by the condition (acquired syphilis) and those resulting from other causes, in lines 219 – 222.

**Answer to Reviewer #3**

**The introduction revels lack of knowledge about syphilis. The data and its analysis do not support the conclusions. There is no way to check the quality of the reported cases, no data about trends on use of public services and no prevalence data.**

We respectfully disagree that the introduction reveals a lack of knowledge about syphilis. We reaffirm syphilis as a serious public health problem; we present definitions, forms of transmission of the disease and forms of treatment. We describe that the disease manifests itself in elderly individuals, a fact that has been demonstrated since the last century. We talked about the incidence of the disease in the world; we emphasize the importance and concern of the infection reflected in a pandemic, and we present notification data and detection rate in Brazil. Lines 105 - 111 demonstrate knowledge of syphilis in relation to its manifestation in the elderly public. Finally, we highlight the importance of the development of the study.

Furthermore, we agree that, at times, there may have been confusion within the text. Thus, it was adjusted in some points. In the table below, we demonstrate that the data and analysis are consistent with the conclusions presented.

| **Conclusion based on the data** | **Data and analysis that support the conclusion** | **Line** |
| --- | --- | --- |
| Although the South and Southeast regions concentrate the highest detection rates of syphilis in older people, the temporal analysis research demonstrated a significant increase in the growth trends of syphilis in all five regions of Brazil, affecting both sexes, but mainly white and black individuals and those with low education. | From the calculation of the annual percentage change (APC) and average annual percentage change (AAPC) of syphilis rates in older people, by region of Brazil and according to gender and three age groups (60-69 years; 70-79 years and 80 years or more), it was found that the growth trends of the disease were significantly homogeneous, considering the variables mentioned.  The detection rate of syphilis in Brazil was higher in males (34,74 cases per 100,000 individuals) than in females (18,46 cases per 100,000 individuals).  Regarding their level of education, 3,702 individuals were illiterate (practically, 0.67 in every 100,000 older people); 5,016 revealed having primary education (0.90 in every 100,000 older people); and 5,150 had high school education (0.93 in every 100,000 older people).  In this analysis, white, black, yellow, and indigenous older individuals presented detection rates of 5.45; 5.11; 0.10, and 0.07 cases, per 100,000 people, respectively. | Lines 201 - 203  Lines 244 - 248  Lines 183 - 186  Lines 244 - 248  Lines 194 - 198  Lines 191 - 192 |
| Inequalities in syphilis care are directly related to individual socioeconomic and demographic characteristics. | This study sought to characterize, in the most reliable way, the epidemiological profile of older people infected with acquired syphilis who are users of the public health network.  The main variable considered for characterizing the epidemiological profile was the diagnosis of acquired syphilis in individuals aged 60 years or more. The independent variables corresponded to those of sociodemographic interest: the reporting of the first symptom onset; age group (60 – 64, 65 – 69, 70 – 74, 75 – 79, 80 – 84, 85 – 89, and 90 or more); gender (male and female); race/color (white, yellow, indigenous, and black); level of education (illiterate, primary, secondary, and higher education); region of the country in which the federative units are grouped; and classification of syphilis according to the cure or death statuses.  The entire topic of the results presents the data referring to these variables. | Lines 178 - 180  Lines 178 - 279 |
| The increase in the detection rate of STIs among older people alerts to changes in sexual behavior and needs regarding health promotion, such as guidance on safe sexual practices and commitment to early diagnosis and treatment. | When analyzing the detection rate of syphilis in Brazil, between 2010 and 2020, a progressive increase in this STI has been observed in older people since 2011, which intensified in 2018. | Lines 181 - 183 |

Concerning the quality of reported cases, it should be noted that the aforementioned study works with a national database - Information System for Notifiable Diseases - SINAN. This database is mainly powered by the notification and investigation of cases of diseases and injuries under compulsory notification (Consolidation Ordinance No. 4, September 28, 2017). Its use allows the realization of a dynamic diagnosis of the occurrence of an event in the population, by providing subsidies for causal explanations of such diseases, in addition to indicating risks to which people are subject, thus contributing to the identification of the epidemiological reality of a given Geographical area. Its systematic use, in a decentralized way, allows all health professionals to have access to its information and make it available to the community. It is, therefore, a relevant instrument to assist in health planning and define and evaluate the impact of intervention priorities (SINAN - Notifiable Diseases Information System. Available at <https://portalsinan.saude.gov.br/>).

Among the limitations of the system, one can mention the high quantity of gaps in fields related to essential variables, such as education and race/skin color, diseases, and associated injuries, in addition to fields referring to treatment. The permanence of records without closure and the non-updating of variables related to laboratory tests are also noteworthy. Thus, the analyzes generated from existing data may not be a good representation, hindering the management of surveillance activities. Thus, the need for a systematic assessment of the quality of the information collected and entered is evident, especially when entering data into SINAN. Operational errors are also highlighted, due to lack of knowledge. The low maintenance capacity should also be highlighted, reflected in the observation of technical failures in the functionality of the system, compromising the analysis of the database, monitoring, and evaluation of the actions conducted. One can also mention the obsolete language and the lack of interoperability with other health information systems. In addition, SINAN does not always update the data collected by the notification/investigation forms promptly (Profile of National Health Databases. Epidemiol. Information System for Notifiable Diseases (SINAN): main characteristics of notification and analysis of data related to tuberculosis. Serv. Saúde 29 (1); 2020. <https://doi.org/10.5123/S1679-49742020000100009>).

With regard to questioning the presentation of data on trends regarding the use of public services, it was not clear how this information would fill any pending gap in the study.

Regarding information on the prevalence data, they were removed at the suggestion of the reviewers themselves, in the previous correction. Thus, we focused on the detection rate of syphilis.

**Answer to Reviewer #4**

**The topic is relevant to the public health area and presents important aspects for evidence-based practice by addressing a sexually transmitted infection in the elderly population. Despite the relevance and excellence in the preparation of the manuscript, it has weaknesses that put in check the uniqueness of the manuscript, since there are national publications on the subject with data from 2022 and the manuscript presents a temporal limitation with the use of data only until 2020.**

In a literature search, we did not find a manuscript that addressed the trend analysis and demonstrated the temporal evolution of acquired syphilis in elderly individuals in Brazil. We believe that existing national publications with data from 2022 did not assess the evolution trend of syphilis in elderly people. In addition, the epidemiological bulletin for the year 2023 is only published with data for 2022, in October. If researchers are using national data for this period, they are probably not referring to corrected data.

Regarding the question on the period of data use, the manuscript presents information up to 2020, as the database provided at the time of its construction presented complete data up to that year, corrected and consolidated. Data for 2021 was still incomplete. In addition, looking at the data for 2020, it was observed that the Covid-19 pandemic influenced the reduction in the number of notifications of cases of acquired syphilis, as well as at the beginning of the following year.

**The study design is questionable, since modern epidemiologists such as Lash et al. Modern Epidemiology present scientific evidence for such aspects. However, the study also has a high descriptive potential, and the statistical analysis used constitutes only one of the first analyses of the composite possibility in the context of time series that are naturally defined as ecological study.**

Ecological studies analyze a group of individuals, using collective measures (aggregated according to geographic or temporal factors). There is no way to know who was sick or exposed, only the total of each group (exposed and unexposed). In this type of study, the research problem refers to global data, in the form of social and health indicators, of aggregates/collectives of individuals. This survey does not work with aggregated data. Thus, the cross-sectional study represents the best methodology for this study. This is because such studies are indicated when one wants to know the prevalence or detection of a certain outcome, as well as the factors associated with this distribution in a defined space, time, and population (MEDRONHO, Roberto A. (Ed). Epidemiology 2. ed. São Paulo: Atheneu, 2009. 685p. ROUQUAYROL, Maria Zélia; SILVA, Marcelo Gurgel Carlos da.(Orgs). Epidemiology & health. 7. ed. Rio de Janeiro: Medbook, 2013).

**Nevertheless, the categorization of the age range is also questionable, as there is no evidence of age up to 120 years, which raises the questioning of the quality of the data.**

There is a possibility that there is a mistake due to the age presented in the records. However, we work with a national database and the information obtained when the individual is asked directly about their age is automatically recorded. Furthermore, as mentioned above in the limitations of this study, it should be taken into account that working with secondary data may imply the presence of intercurrences such as operational errors. On the other hand, it is increasingly necessary that these systems be used properly so that they can be perfected.

In order to reaffirm the limitations and respond to the queries from the reviewers regarding the use of the SINAN database, we have added some information within the body of the article (also included in this response letter).

**The discussion presents relevant points, but little expressive regarding the importance of completeness and completion of official documents of notification of diseases that should be exalted in the limitations of the study.**

As suggested, in the limitations of the study we reaffirm that there is incompleteness of data mainly regarding elderly people (linhas 414 – 422).

**Another important point is that despite understanding that the data are of free access, it should be made clear that the research was not submitted for ethical review.**

Recommendation accepted, as shown in lines 127 -128.

**Finally, despite the relevance of the theme and quality of the writing, I recommend that the authors make the necessary adjustments and submit the manuscript for publication in a public health section.**

While responding to queries from the reviewers, we did our best to address the proposed new recommendations. At the opportunity, we thank you for the guidelines sent and reinforce our desire to publish in a mega journal such as PlosOne.
