# Supplementary material for: Acquired syphilis in older people in Brazil from 2010–2020
Source: PLoS One. 2024 Sep 6;19(9):e0296481. doi: 10.1371/journal.pone.0296481 (PMC11379233; doi:10.1371/journal.pone.0296481)
Supplement: Attachment — Submitted filename: Response to Reviewers.docx [file pone.0296481.s004.docx]

**Answer to the reviewers**

**Editor's suggestions**

1. **Writing Clarity**:

- Please enhance writing clarity, refine sentence structure, and minimize the use of parentheses for a more fluid narrative. Additionally, ensure the use of bias-free language throughout your manuscript.

We sought to respond to the editor's suggestions in the best possible way.

2. **Supplemental Files**:

- Ensure that supplemental files are appropriately cited in the document and adhere to the correct naming conventions.

Suggestion answered.

- Please exclude the list of figures as a supplemental file.

Suggestion answered.

- The supplemental file "notification_acquired syphilis" is currently in Portuguese. Please also include an English translation for this file.

As it is a Brazilian government information system, there is no English version.

- The remaining supplemental files are currently named in Portuguese, and their content is not clearly specified. To align with the publication standards, it is advisable to rename these files in English and provide concise descriptions to elucidate their respective contents.

Suggestion answered.

**Abstract**

- Please address the temporal discrepancy in the abstract, where main findings refer to data up to 2018 while the research scope extends to 2020.

We have implemented the suggestion on lines 43-44.

**Introduction**

- It would be beneficial to include additional references when stating "Some studies highlight a high prevalence of these infections in individuals over 60 years, including those in extreme age groups"

As suggested by the reviewer, references were added in lines 104-105.

- Consider substituting the figurative language "invisible" in the statement " structural ageism, making their sexuality invisible."

As requested, the correction was performed on line 114-115.

**Methods**

- Please include a link to the database when referencing the data source.

Suggestion answered: lines 130; 232; 252; 260; 282.

- Clarify the statement "It is essential to point out that not all information contained in the SINAN notification form is made public" particularly regarding the transparency of the data presented in this manuscript.

To avoid any interpretation problems, we removed the phrase.

- Consider using "was obtained" instead of "came from" in the statement "The information regarding these variables came from the SINAN database."

As requested, the correction was provided on line 180.

**Results:**

- Please provide clarification regarding the statistical significance of the observed difference mentioned in the statement "It was higher in males (34.74 cases per 100,000 individuals) than in females (18.46 cases per 100,000 individuals)."

The sentence was rewritten as suggested by the reviewer, to avoid misinterpretations. Lines 224-226.

- Consider changing the caption of Fig 2, reflecting the prevalence of acquired syphilis in older people among different regions of Brazil.

The caption was changed as requested.

- Kindly note that Fig 3 contains words in Portuguese. Please ensure compliance with language standards.

Correction implemented in Fig 3.

- Provide an explanation for the use of '*' in some numbers in Table 1.

As requested, the explanation has been implemented.

**Discussão:**

- Please correct the capitalization of "Covid-19" for precision

As requested, the suggestion was implemented on lines 348-349.

- Prioritize the interpretation of data before drawing comparisons with other studies. Additionally, always describe the statistical significance when stating that a particular statistical pattern is more or less pronounced.

We chose to use confidence intervals because they offer a richer and more informative way of presenting statistical results, widely used in the literature on trend analysis. In addition to statistical significance, confidence intervals evaluate the magnitude of the result, allowing the reader to understand beyond the specific information of a p-value. The Confidence Intervals presented in Table 1 contain information about the point estimate and variability of the underlying data, while p-values generally only provide information about statistical significance. However, to comply with the request, we have included a column with the p values.

**Reviewer #5:**

- Several points of the introduction must be improved (see Major Compulsory Revisions). Moreover, I do suggest updating the references by referring to recently published major articles in Infectious Diseases as Peeling RW, et al. Lancet. 2023. doi: 10.1016/S0140-6736(22)02348-0 (see Major Compulsory Revisions)

As requested, suggestion implemented on lines 65-67; 69-71; 83-85; 87-89; 100-105 and 106-109 of the introduction.

- Please, at least at the beginning of the text, try to correctly address the name of the pathogen which is Treponema pallidum (in italics).

As requested, the correction was implemented on lines 54; 69 and 77-78.

- The authors state that “the primary and secondary types are easy to diagnose”. I appreciate the numerous historical events cited by the authors: considering this, the authors should know that syphilis has been always considered “the great imitator” by clinicians. Syphilis is not an easy disease to diagnose. Not only for its various clinical features, but also for the extremely complex interpretation of non-treponemal titers [Marchese V, et al. J Clin Med. 2022. doi: 10.3390/jcm11247499]. Please, try to mitigate your statement, and try to expand the clinical presentations by referring to the extremely wide differential diagnosis [Tiecco G, et al. Pathogens. 202. doi: 10.3390/pathogens10111364].

As requested, the suggestion was implemented in lines 69-75; 108-109; 76-77.

- Please, try to update epidemiologic data from all over the world by referring to recently published major articles in Infectious Diseases [Peeling RW, et al. Lancet. 2023. doi: 10.1016/S0140-6736(22)02348-0].

As suggested, the epidemiological data were updated in lines 77-98.

**Reviewer #6**

- The conclusion in the abstract is directly supported by the result, while the conclusion from lines 437 to 446 are not supported by this study, more like for discussion.

As suggested by the reviewer, we have adjusted the conclusion of the study in lines 483-491.

- There is lack of the details how the case was defined/diagnosed, how cure/death was tracked and how some detection rates were calculated. To understand the significance of increased detection rate in elders, it should be compared with the change of detection rate in younger adults.

Throughout the text, we sought to meet, in the best possible way, these considerations. The details were highlighted in the topics relating to each request.

- If the detection rate in younger adults was similarly or more increased, then the public health should remain focused on younger adults.

Although the syphilis detection rate also increases in individuals under 60 years of age, we must be concerned about older individuals, as it is observed that this population is increasingly included in the risk group for sexually transmitted infections. Furthermore, the number of older individuals is growing worldwide, requiring more and more diverse health care.

- Introduction is very long and should be more focused.

As requested by the majority of reviewers, the entire introduction was reorganized in order to meet the suggestions.

- Paragraph 3 can be shortened to one sentence.

As suggested by previous reviewer, paragraph 3 has been removed.

- Paragraph 4 is not relevant other than penicillin.

As suggested by another reviewer, paragraph 4 was also removed.

- Remove 84 a 85.

Not only were the suggested lines removed, but also the paragraph, replacing it with others containing updated information: lines 77-89.

- Give more introduction of SINAN, either in the introduction part or in Materials and Methods, such as who is doing the reporting, how it is reported and what is the reporting criteria?

Suggestion implemented on lines 131-164.

- How to ensure all cases are reported consistently over the years. The study is performing the temporal trend analysis, that could be invalid if the reporting criteria has been changed over the years.

As this is a compulsory notification system, all people will be included in the SINAN by the health professional who provided care to that patient, as per Ministerial Ordinance – Consolidation Ordinance N°. 4, of September 28, 2017. Lines 151- 157.

- As introduced between lines 99 and 100, the improvement of reporting may explain the increase in syphilis cases in Brazil. Is there data to describe the improvement of reporting, such as the potential population covered by the reporting system? This is useful to understand the increased detection rate, that is based on geographic census. As indicated by authors in the reply, the information of disease stage is not available, this should be specified in Materials and Methods.

Professionals are constantly updated to ensure the effectiveness of compulsory notification in SINAN. In this way, improving the notification record is achieved by eliminating subrecords. With the interoperability of information systems, it is possible to identify the potential population covered by the geographic census, and thus, identify risk groups. From there, strategies are developed to attract these individuals to health services, or carry out an on-site search, and thus track the presence of syphilis through testing, consequently notifying and treating these patients.

Regarding information on the stage of the disease, this data is not available in the database since it is difficult to classify the type of syphilis. Therefore, the information is not published to avoid bias.

In the text, the suggestions were implemented in lines 435-444.

- Lines 135-137, need more clarification to understand ‘therefore’ and ‘secondary data’? Is this a portion of data since 2010?

Information on syphilis was used, collected from the official public health database system of the Brazilian Ministry of Health. The “Mandatory Notifiable Diseases Information System” - SINAN is the main source of secondary data available from the Brazilian notification system, as all cases of the disease are notified and registered in this system.

As requested, the suggestion was implemented on lines 175-178.

- Line 140-141, unclear the criteria for the diagnosis of acquired syphilis.

Suggestion implemented on lines 159-164.

- Lin 146-147, unclear how the reporting system works and how to track the cure and death history.

Suggestion answered on lines 165-172 and 142-145.

- Line 132-133 and Line 153-155 are both about detection rate calculation. Please combine.

Information about detection rate calculation was removed from lines 132-133 and kept in lines 193-195.

- Line 157-158, not clear what ‘constant’ was used and why?

To obtain the detection rate result, the calculations are standardized per 100,000 – the constant used. As requested, the suggestion was answered on line 198.

- **Results**
- Line 182 in, need to specify the age group of older people to avoid confusion.

As requested, the suggestion was answered on lines 222-223.

- Line 184-185, not sure how 12.84 is derived? It is not implicated in fig 1 and does not fit with male and female results (34.74 and 18.46). Is it the journal style to use ‘34,74’ cases instead of ’34.74’? It is more confusing when reading the fig 1 and fig 5 with ‘,’ instead of ‘.

We understand the doubt, but detection rates should not be added together, that is, the sum of male and female detection rates should not be considered and compared with the final detection rate. The proportion is calculated. Below is a description of the calculation for detection rates:

Total female older individuals in the database: 28577

Total female older individuals in Brazil in the analyzed period: 154789425

**Detection rate for females** = 28577 x 100.000 = 18.46

154789425

Total male older individuals in the database: 42674

Total male older individuals in Brazil in the analyzed period: 122824712

**Detection rate for males =** 42674 x 100.000 = 34.74

122824712

Total missing information in Brazil: 38

Total of older individuals of both sexes: 277614137

**Unreported data detection rate =** 38 / (154789425 +122824712) x 100.000 = 38 / 277614137 x 100.000 = 0.01

**Final detection rate** = (28577 + 42674 + 38) / (154789425 + 122824712 + 122824712) x 100.000 = 71289 / 555228274 x 100.000 = **12.84**

As requested, the correction was made regarding the use of “.”, instead of “,” in graph 1. Graph 5 was removed.

In fig 1, what is first symptoms?

Unfortunately, information about first symptoms is not available in the database.

- What is difference between fig 1 and fig 5. There is some difference but very small.

We agree with the opinion regarding the difference between the graphs, and to avoid redundancy, it was decided to remove Fig 5 and keep Fig 1.

- Need more introduction in Materials and Methods or in figure legends.

In materials and methods, more information was added as suggested by the reviewers.

- In both fig 1 and fig 5, please include the rate change among younger adults. This will help the readers to understand the significance of increased detection rate among elders.

As it is beyond the objective of our study to compare the variation in the syphilis detection rate between younger adults and older individuals, we tried to comply with suggestions from the reviewer, in a certain way, in the discussion, where we included updated data on syphilis detection rates in younger individuals (lines 351-358).

- Line 194-200, calculation of % in every 100,000 older people is misleading, if it is not adjusted for % of each level of education in every 100,000 older people. In other words, calculate detection rate in every 100,000 illiterate older people, or every 100,000 older people with high school education… Such information is required for later discussion in line 357-362.

As advised by the reviewer, the education level information was transformed into percentage values. The number of elderly people with syphilis in the analyzed period, according to the educational level category, was divided by the total number of elderly people in Brazil, in the same period of the trend analysis, and then multiplied by 100. The results can be seen in the lines 238 – 242.

- Line 202-203, not sure the meaning of ‘even higher than the national rate’. Certain regions are expected to have rates higher than national rate, if the rate is not same across different regions. Does it mean national rate in total population? Please specify.

It refers to the total population of older individuals. It is specified on line 247.

- It is better to show the detection rate in total population or younger adults in each region.

This suggestion is very pertinent, but an analysis of the detection rate in the total population or in younger adults is beyond the objective of our study, which prioritizes the analysis of the detection rate in elderly individuals. We can even consider, as future work, carrying out a study that compares the detection rate between younger adults and older individuals.

- Line 210-211 and fig 3, the comparison should include the younger adults from 18 to 60, ideally split to different age sub-groups. This will help to understand the significance of the detection rate among elders.

We appreciate the suggestion, but if we were to compare the rate of syphilis between older and younger individuals, we would have to change the objective of our study. On the other hand, we included the detection rate of syphilis in younger individuals in the discussion (lines 335-336; 345-358), to respond to the request to present data to get an idea of the detection rate in individuals under 60 years.

- Line 45-46 should have the same context to indicate that the mean age of 68.04 years is for adults aged 60-120.

As requested, the suggestion was implemented on lines 45-46.

- Line 216-222 and fig 4, without the introduction of how disease was tracked and how cure or death was reported, it is difficult to understand this part, especially in the absence of disease stage information. Also need to relatively compare with the changes in younger adults in order to justify the significance of changes in elders.

In materials and methods, we incorporate more information related to disease screening, as well as reporting on cure or death. We hope we have complied with the suggestion. Lines 168-172 and 442-444.

Regarding the comparison of changes in younger adults with older ones, it is beyond the scope of our study to address this at the moment, which prioritizes the discussion of acquired syphilis in the population of older individuals.

- Line 285-287, 298-305 should be moved to introduction or Materials and Methods.

Suggestion implemented on lines 132-134; 135-145.

- **Discussion**
- Line 326-332, not sure whether the difference in white and black is significant in this study. Does not seem to be relevant.

As we brought updated data on syphilis rates in the older population, until 2022, depending on race/color, we decided to keep the information. Lines 379-382.

- Line 336-337, did not address previous reviewer comment, need proof, such as a reference. Or correlate with other cited references from line 339-345.

We have implemented the suggestion on line 361.

- Line 346-350, please discuss why the cited reference is relevant or different with this study, and what is the implication.

When restructuring the discussion, the paragraph and consequently the reference cited, appears in the text with another type of implication. Lines 393-396.

- Line 351-352, similar to reference 30, please compare with the detection rate in younger adults in order to discuss the significance of increased detection rate in elders.

Suggestion implemented on lines 351-358; 371-373 and 452-457.

**Reviewer #7:**

**Abstract**

- Background: I suggest the authors bring the syphilis concern among older people.

Suggestion implemented on lines 29-31.

- Methodology: consider the appointments below.

All the suggestions for this topic were implemented.

- Conclusion: The conclusion should be focused on your results on older people.

Suggestion implemented on lines 49-51.

**Introduction**

- Page 3, line 56- “…although it was discovered in the late 1400s [2]”.
  The sentence and reference two need to be more appropriate. This is a secondary reference that discusses the origin of Syphilis, not the discovery.

As requested, the suggestion was implemented on lines 55-56.

- Page 3, line 61- Please review reference 4 in the context of this sentence.

After reviewing the reference information, line 61 has been removed.

- I suggest deleting lines 68 to 77 and 78 to 83. This information is unnecessary.

The suggestion was duly addressed.

**Material and Methods**

- This is an ecological study because the unit is “time”, despite the authors using public micro or macro data!

As suggested, the correction was implemented on lines 35 (abstract) and 122.

- Line 137. Is there any case aged 120 years?

In the database used, during the period evaluated, there are records of 26 cases of individuals aged 120 years.

- The authors should show the characteristics of Brazil to the readers: number of regions, number of inhabitants, number of people aged 60 years and over, human development index, etc.

Suggestion implemented on lines 123-127.

- Also, they need to describe clearly how the Brazilian system defines acquired syphilis.

Suggestion implemented on lines 54-55; 56-57 and 151 -164.

- Page 6, line 114. Is there someone aged 120 years?

In the database used, during the period evaluated, there are records of 26 cases of individuals aged 120 years.

- Please present the percentual of missing data for each variable.

It was more pertinent to present the percentages of lost or missing data in the results. Suggestions implemented on lines 226; 236; 241; 273; 275-276.

In addition to presenting the percentages of missing data, as requested, in the discussion (lines 340-346) the problem of not filling in this information was addressed, and the limitations of the study were also discussed - lines 493-501.

- Joinpoint Regression – please include the reference of the software

Suggestion implemented on the lines 209.

- **Results**
- Table 1 presents data only for North, Northeast, and Mid-West Regions! In addition, there is no legend! Please review it.

As requested, corrections were made to Table 1.

**Discussion**

- Line 292. It would be best if you were more precise. What do you mean about it? Underreporting of death and cure? Or underreporting of syphilis diagnosis? Or underreporting of syphilis diagnosis.

As suggested by the reviewer, the information was made clearer in the third paragraph of the discussion - lines 329-334.

- Line 293. I suggest replacing “manifestation of the disease” with “notification of the disease.”

The paragraph was restructured on lines 345-350.

- Linha 298-310.  I suggest moving this information to Material and Methods.

The suggestion was implemented on lines 135-150.

- Line 311 to 317. If the decrease in syphilis cases is due to COVID-19 (and very likely is), then it should be desirable to evaluate the syphilis trend forward.

This suggestion for future work to be developed is extremely important. On the other hand, we present how the disease detection rates behaved in the country for the elderly, after the pandemic period, on lines 356-358.

- Lines 322 and 323 – “On the other hand, the increase in testing coverage and reorganization of health units did not stabilize the upward curve of the disease.”
  It is noteworthy, according to your data, in 2019, before the COVID-19 Pandemic, there was a slight reduction in cases. This could be a trend-forward that was interrupted due to the pandemic.

After the pandemic period, reported cases increased again, according to the 2023 Epidemiological Bulletin. The evolution of the number of cases can be observed in lines 345-350, through the data presented in the latest epidemiological bulletins.

- Linhas 336-337 – Please provide a reference.

Suggestion accepted, as requested by the reviewer. Line 361

- Linhas 339-345 – Bernstein et al. is not an appropriate reference. The discussion is about the higher syphilis frequency among men vs. women. Does it occur in Brazil? Why does it happen? I think you should also compare your data with other Brazilian studies.

As suggested, the reference to Bernstein et al. was revoved.

Yes. Between 2012 and 2022, it was observed that 750,848 (42.3%) cases of acquired syphilis occurred in men and 485,115 (47.4%) in women. According to the suggestion, the articles were replaced by studies in Brazil in lines 361-363; 364-367 and 371-373

- Lines 347-350- I didn’t understand the references 28 and 19 in this context! Please review it.

In agreement with the reviewer, it was decided to remove the paragraph.

- Lines 351 – 356 – Please discuss your data about syphilis. Syphilis among older people!

Suggestion implemented on Lines 422-424; 445-451.

- Lines 371-375 – I think this paragraph is loose!

In agreement with the reviewer, it was decided to remove the paragraph.

- Lines 376 e 381 - Provide references. In Brazil? Please rewrite this paragraph more clearly.

As the discussion already presents sufficient information also contained in this paragraph, it was decided to remove it.

- Lines 384 a 389 – Despite the data's fragility, they showed a high cure rate for syphilis. Discuss it!

As suggested by the reviewer, the paragraph was rewritten to make it clearer in lines 402-407.

- I suggest an extensive English review.

Suggestion accepted
